# Outcomes and early revision rate after medial unicompartmental knee arthroplasty: prospective results from a non-designer single surgeon

**DOI:** 10.1186/s12891-018-2099-2

**Published:** 2018-05-29

**Authors:** Jonathan R. B. Hutt, Avtar Sur, Hartej Sur, Aine Ringrose, Mark S. Rickman

**Affiliations:** 1 Department of Trauma and Orthopaedics, St George’s University Hospitals NHS Foundation Trust, London, UK; 2 Department of Orthopaedics and Trauma, The University of Adelaide and Royal Adelaide Hospital, Adelaide, Australia

**Keywords:** Unicompartmental, Arthroplasty, Outcomes, Survivorship, Indications

## Abstract

**Background:**

This prospective study evaluates outcomes and reoperation rates for unicompartmental knee arthroplasty (UKA) from a single non-designer surgeon using relatively extended criteria of degenerative changes of grade 2 or above in either or both non-operated compartments.

**Methods:**

187 consecutive medial mobile bearing UKA implants were included after history, clinical assessment and radiological evaluation. 91 patients had extended clinical outcomes. Post-operative assessment included functional scoring with the Oxford Knee Score (OKS) and radiographic review. Survivorship curves were constructed using the life-table method, with 95% confidence intervals calculated using Rothman’s equation. Separate endpoints were examined: revision for any reason and revision for confirmed loosening.

**Results:**

The mean follow-up was 3.5 years. The pre-operative OKS improved from a mean of 21.2 to 38.9 (Mann-Whitney U Test, p = < 0.001). Twelve Patients required further operations including 9 revisions. No patients developed deep infection and no surviving implants were loose radiographically. Survivorship at 7 years with endpoints of re-operation, revision and aseptic loosening at surgery or radiographically was 88.4% (95% CI 79.6–93.7), 93.1% (95% CI 85.5–96.9) and 97.3% (95% CI 91.2–99.2) respectively. The presence of pre-operative mild contralateral tibiofemoral or any extent of patellofemoral joint degeneration was of no consequence.

**Discussion:**

The indications for UKA are being expanded to include patients with greater deformity, more advanced disease in the patellofemoral joint and even certain features in the lateral compartment indicative of an anteromedial pattern of osteoarthritis (OA). However, much of the supporting literature remains available only from designer centres. This study represents a group of patients with what we believe to be wider indications, along with decisions to treat made on clinical grounds and radiographs alone.

**Conclusion:**

This study shows comparable clinical outcomes of UKA for extended indications from a high volume, high-usage non-designer unit.

**Electronic supplementary material:**

The online version of this article (10.1186/s12891-018-2099-2) contains supplementary material, which is available to authorized users.

## Background

The Oxford unicompartmental knee arthroplasty (UKA) (Biomet, Warsaw, Indiana) is a well-established implant and reports from the designer centre demonstrate good results for the medial UKA out into the second decade [1] and into the midterm for its lateral counterpart [2]. Whilst the initial indications were relatively narrow, increased experience has led to an expansion of potential inclusion criteria, particularly with regard to the level of deformity and disease presence elsewhere in the knee [3–5]. The performance of the Oxford UKA in the wider orthopaedic community has been variable, with units reporting conflicting results, some equally favourable [6–10], and others less so [11–14]. Much of the concern regarding UKA in general has come from the analysis of registry data. The Australian, the New Zealand and the UK implant registries all report higher revision rates for unicompartmental prostheses [15–17]. There is a debate as to what registry data can reveal about the success of an implant or technique, and analysis of published literature on the subject will be biased by numerous reports from the designing centre [18–20]. As such, reports from surgeons independent of such centres add valuable information on outcomes from the use of implants by the wider orthopaedic community.

Between 2005 and 2013, the senior author implanted 187 consecutive UKAs in 173 patients, a caseload of 23 per year. During the same period, the senior author performed 604 TKAs, and 12 lateral UKAs. 14 bilateral UKA procedures were performed sequentially. This corresponds to a usage of 30% in keeping with recommendations that 30% of a surgeon’s total knee arthroplasties should be UKAs to achieve optimum results [7, 21, 22].

The aim of this study was to prospectively evaluate the early outcomes and revision rate from a single high volume non-designer practice of unicompartmental knee replacement as well as the effect of using relatively extended criteria with regards to other compartments in the knee.

## Methods

Patients presenting to the senior author with symptomatic knee arthritis are evaluated for their suitability for UKA as follows: The history and clinical examination focuses on presence of isolated unicompartmental knee pain severe enough to justify joint replacement and anterior cruciate ligament (ACL) integrity. Clinical evidence of sagittal instability and the presence of inflammatory disease remain absolute contraindications to UKA. Maximum acceptable pre-operative deformity is 15 degrees of varus that is correctable to neutral and 10 degrees of fixed flexion. No patients have been refused UKA based on BMI. Radiographic evaluation is with standing AP and Rosenberg views along with standard lateral and skyline views. The presence of bone-on-bone contact was considered an indication to proceed with UKA. Stress radiographs and MRI scans are not used. Evidence of mild disease of the contralateral compartment, for example marginal osteophytes, is not considered a contra-indication in the setting of minimal joint space narrowing. Degeneration of the patellofemoral joint (PFJ) is considered irrelevant unless pain is wholly anterior, and specifically worse on stairs than with simple walking. No arthroscopic examinations are performed solely to evaluate the knee for decision-making purposes. For this study, both the patellofemoral and contralateral tibiofemoral compartment were evaluated on pre-operative radiographs according to the Kellgren-Lawrence grading. We considered patients with evidence of degenerative change of grade 2 or above in either or both non-operated compartments to have relatively extended indications for UKA. At surgery, ACL integrity is assessed clinically with examination under anaesthesia (EUA) and direct inspection and the lateral compartment is also directly inspected. Intraoperative findings of patellofemoral joint degeneration, whatever the severity, are not considered a contraindication to UKA. Within the time frame of the study, no patients were converted to TKR based on concerns with ACL integrity at operation. Three patients scheduled for a UKA received a TKA due to significant lateral disease that was not identified on pre-operative radiographs. Surgical technique was per manufacturer guidelines using a tourniquet and thigh support with free draping of the limb using the described minimally invasive approach [23]. All patients underwent a standardized post-operative recovery physiotherapy programme of immediate full weight bearing, range of motion and strengthening exercises without restrictions. Post-operative review was at 6 weeks, 6 months and then annually, including functional scoring with the Oxford Knee Score (OKS) and radiographs with standard AP standing and lateral views. All patients were followed prospectively and reviewed by independent examiners. For this study, patients undergoing combined UKA and ACL reconstruction have been excluded.

### Statistical analysis

Data was tested for normality using D’Agostino’s K^2^ test. Pre-and post-operative OKS were thus compared using the Mann-Whitney U-test with significance set at *p* < 0.05. Correlations for age and BMI used Spearman’s rank test. Complication rates for extended indications were compared using Fisher’s exact test. Survivorship curves were constructed using the life-table method, with 95% confidence intervals calculated using Rothman’s equation [24, 25]. Separate endpoints were examined: revision for any reason and revision for confirmed loosening. Patients who died or were lost to follow-up were treated as censored data.

All procedures performed in studies involving human participants were in accordance with the ethical standards of the Clinical Research Facility of St George’s Hospital.

All patients provided written informed consent to their data being part of this study as part of their surgical consent.

## Results

**Fig. 1:**
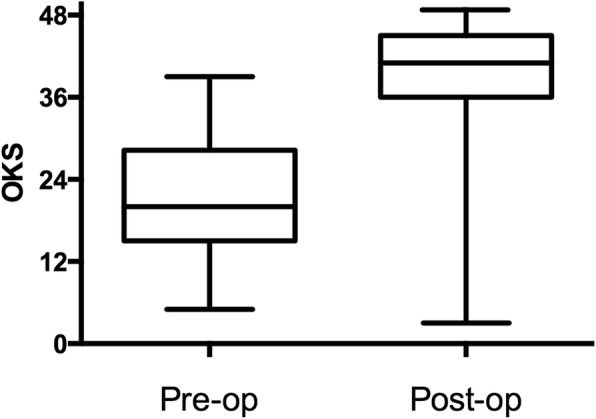
Pre-and post-operative OKS

Between 2005 and 2013, the senior author implanted 187 consecutive UKAs in 173 patients. During the same period, the senior author performed 604 TKAs, and 12 lateral UKAs. 14 bilateral UKA procedures were performed sequentially. Patient demographics are shown in Table 1. The mean overall follow-up was 3.5 years. 5 patients died from unrelated causes, all with well-functioning implants. 7 patients (3.7%) were lost to follow-up and proved untraceable. 2 had data at 6 months, whilst 5 had no follow-up data available. The pre-operative OKS improved from a mean of 21.2 to 38.9 (Fig. 1, p = < 0.001). There was no correlation between the post-op OKS and either age (*p* = 0.88) or BMI (*p* = 0.47).

**Table 1:** Cohort Demographics

	UKA Patients
Number	187
M:F	92:95
Mean BMI, Range	29.7 (17.9–45.1)
Mean Age at Surgery / Years, Range	64.2 (49–84)
Mean Follow-up / Years, Range	3.6 (0.5–8)

Twelve patients required 13 further operations. Two required bearing revision after dislocation within 6 months. One of these was later revised at 7 yrs. for progression of osteoarthritis (OA) in the lateral compartment, whilst the other had no further problems. Four patients had revision to total knee arthroplasty (TKA) for pain alone, without evidence of component loosening at surgery. Three patients had revision for femoral component loosening, all with single peg components. Two were converted to TKA and 1 had a revision of the UKA femoral component alone to a twin peg design. One patient was revised to a TKA for progression of arthritis following multiple haemarthroses for a familial bleeding disorder. Two further patients had additional lateral compartment and patellofemoral arthroplasty respectively without revision of the original UKA. No patients developed deep infection and no surviving implants were loose radiographically.

**Fig. 2:**
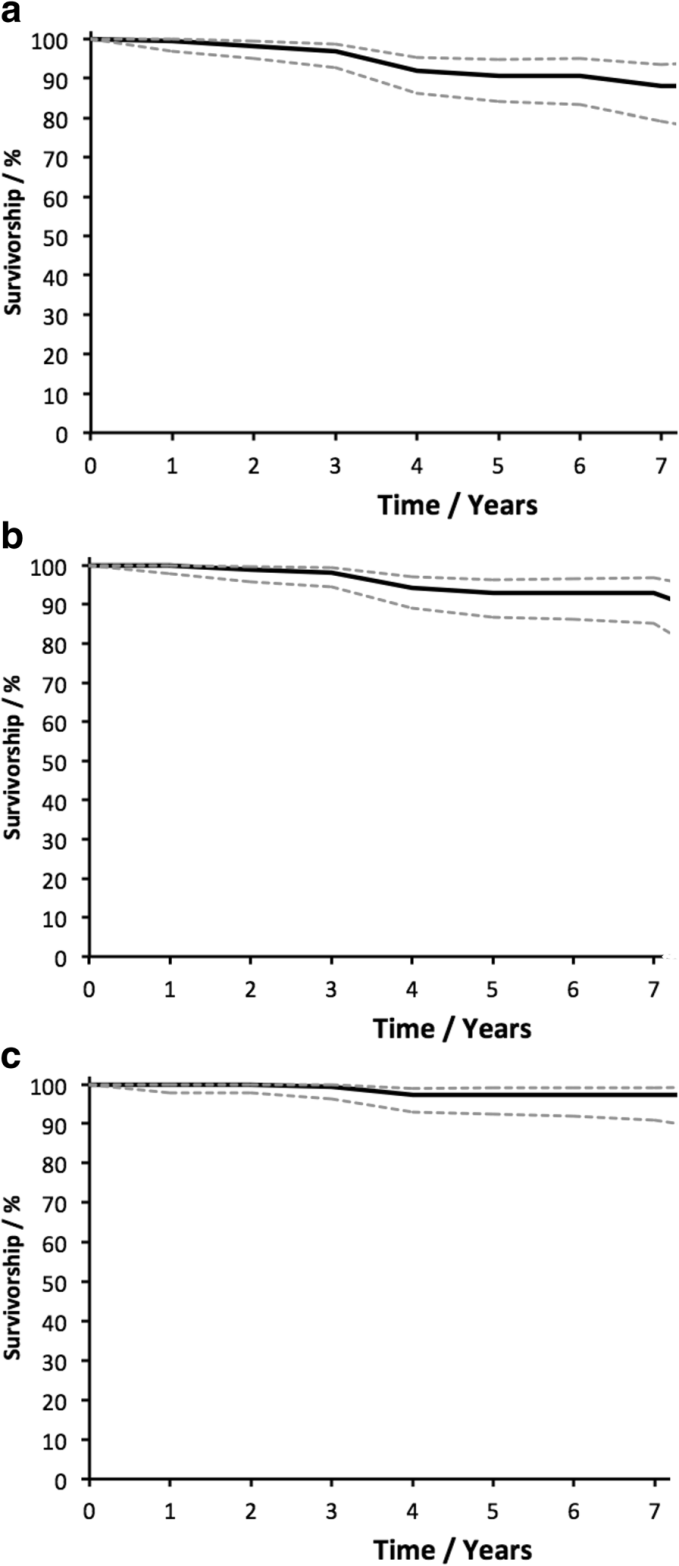
**a** Survivorship curve with 95% Confidence Intervals for Re-operation. **b** Survivorship curve with 95% Confidence Intervals for Revision. **c** Survivorship curve with 95% Confidence Intervals for Aseptic Loosening

Survivorship at 7 years with endpoints of re-operation, revision and aseptic loosening at surgery or radiographically was 88.1% (95% CI 79.1–93.5), 92.9% (95% CI 85.1–96.8) and 97.3% (95% CI 90.9–99.2) respectively. All revisions were included for the re-operation endpoint. Only operations where the UKA implant was removed or replaced were included for the revision endpoint. The full survivorship curves and confidence intervals for the three outcomes are shown in Fig. 2a-c. The complete life tables for each outcome including confidence intervals and effective number at risk each year are provided in Additional file 1.

### Effect of degeneration in other compartments

96 patients (51%) had no pre-operative extended indications, compared with 91 (49%) who did. The outcomes for patients with extended indications in various combinations are set out in Table 2. The extended indications group had a significantly higher OKS (*p* = 0.01), a difference which remained significant for any case with PFJ degeneration (p = 0.01) or with isolated PFJ degeneration (*p* = 0.05). However, as the differences are less than 5 points, an accepted minimally important clinical difference for the OKS, this may not translate into clinical significance. No other comparisons reached statistical significance; importantly, patients without extended indications did not demonstrate superior post-operative outcomes when compared with any subgroups of patients with extended indications including those with a PFJ grade of 3 or 4 (*p* = 0.15). Only one patient with extended indications had a revision to a TKA for arthritis progression – this was the patient with a familial bleeding disorder. Overall, patients with extended indications had significantly lower rates of re-operation and revision (*p* = 0.003).

**Table 2:** Outcomes for Extended Indications

	Number of Patients	Mean Post-op OKS	Re-operation, Revision or Aseptic Loosening
No Extended Indications	96	37.6	12
Either Extended Indication	91	40.3	1
PFJ +/− Lateral grade > 2	93	40.2	0
Lateral +/− PFJ grade > 2	27	40.8	0
Isolated PFJ grade > 2	64	40.1	0
Isolated Lateral grade > 2	3	44.0	1
PFJ Grade III/IV	39	39.5	0

## Discussion

The indications for UKA are being expanded to include patients with greater deformity, more advanced disease in the patellofemoral joint and even certain features in the lateral compartment indicative of an anteromedial pattern of OA [3–5]. However, much of the supporting literature remains available only from designer centres. This study represents a group of patients with what we believe to be wider indications, along with decisions to treat made on clinical grounds and radiographs alone. Only very rarely was the procedure changed based on intra-operative findings.

The senior author implanted 187 consecutive UKAs in 173 patients, a caseload of 23 per year. During the same period, the senior author performed 604 TKAs, and 12 lateral UKAs. 14 bilateral UKA procedures were performed sequentially. This corresponds to a usage of 30% in keeping with recommendations that 30% of a surgeon’s total knee arthroplasties should be UKAs to achieve optimum results Despite the broad criteria and the fact that the senior author receives patients from other consultants in the hospital for consideration of UKA, the ratio of UKA:TKA for patients presenting with symptomatic knee arthrosis in the period of this study was approximately 1:3.

Nine UKA implants have been revised so far, and we are not aware of any currently at risk. Four revisions were for unexplained but persistent medial pain. All were uncomplicated revisions to a TKA using simple primary implants and no obvious cause for pain was identified in any case. Whilst 3 patients have gone on to a reasonable result, one continues to have unexplained pain. None of these patients had extended indications as we have defined. Three femoral components were revised for aseptic loosening. All were of the single peg design, which has been noted in the past to be associated with an incidence of early loosening [26]. One was revised to a twin peg design and continues to function well (recent OKS 47), whilst the other 2 cases were revised to total knee arthroplasty, with satisfactory outcomes. During this series of patients, the twin-peg design for the femoral component became available. Within the literature there are no reports of femoral loosening issues for this iteration. Similarly, there were no failures of the twin peg femoral component in this series. It is possible therefore that these 3 revisions could have been avoided with the use of the newer design implants.

There were 2 bearing dislocations; one undoubtedly due to surgical error, with residual cement at the back of the tibial component leading to anterior dislocation in full flexion. At revision, an identical bearing was replaced after removal of the errant cement. The second dislocation occurred for no clear reason and was revised to a bearing 1 mm thicker; this patient is currently functioning extremely well (2-year OKS 48). The dislocation rate of 1% is in line with other published rates in the literature and remains a potential complication of any mobile bearing UKA design.

Two cases went on to have further compartments replaced – one patellofemoral at 40 months and one lateral at 32 months after UKA. If anything, this represents a failure of patient selection, necessitating a further operation a moderate time after the primary surgery with a rate of 1%. Patient selection and indeed implant selection in any orthopaedic surgery is complex and often difficult, and no selection process will be perfect. By narrowing the inclusion criteria for UKA this 1% failure rate could be lowered, but perhaps not eradicated due to natural variances. There were no deep infections in this series, and this is consistent with other reports of low infection rates for UKA in comparison with TKA [27–29].

For any new orthopaedic implant, favourable results would be expected from developing centres, and whilst it might be rational to assume that similar outcomes will not be achieved by the wider surgical community, our series forms part of a growing number of independent reports of good results and favourable revision rates [6, 7, 9, 10]. There are also reports from other units with less success [11–14], but the main contrast comes from concerns raised primarily by registry data [30]. Inevitably the performance of an implant is dependent on the technique used to implant it, and studies have demonstrated the effect of a significant learning curve for UKA [31–34] with some authors advocating minimum numbers to be undertaken in order to maintain competence [7].

Strengths of the study that should be noted are the series does not include the learning curve of the senior author, with more than 50 procedures being carried out as a registrar and fellow, but all cases carried out as a consultant are included with a usage of 30% as recommended for optimal results.

Limitations include the difficulty to quantify the effect of a single surgeon’s ability to master a new technique. Undoubtedly many surgeons never get over this learning curve before abandoning the technique in favour of either osteotomy or total knee arthroplasty, which will affect global outcomes. Ultimately, the fate of UKA may depend on whether there is any clinical benefit for the patients. Level one evidence on this issue is on the way [30].

## Conclusions

In conclusion, we have shown comparable clinical outcomes and survivorship of the medial Oxford UKA when used with wider indications in a large cohort of patients. Our all cause revision survivorship rate of 93% at 7 years is similar to figures reported from systematic reviews of registry data which show an all cause revision rate for total knee replacement of 6% at 5 years and 12% at 10 years [35]. In addition, the use of extended indications in this series has not had a detrimental effect on post-operative outcomes or re-operation or revision rates. We believe this data justifies the continued use of UKA at our institution within our current indications and serves to highlight the importance of practice analysis by individual surgeons of techniques that might be controversial in the wider orthopaedic literature.

## Additional file

Additional file 1: Outcome life tables. (TIFF 3853 kb)
