# Supplementary figures and images for: Outcomes and early revision rate after medial unicompartmental knee arthroplasty: prospective results from a non-designer single surgeon
Source: BMC Musculoskelet Disord. 2018 May 29;19:172. doi: 10.1186/s12891-018-2099-2 (PMC5975526; doi:10.1186/s12891-018-2099-2)

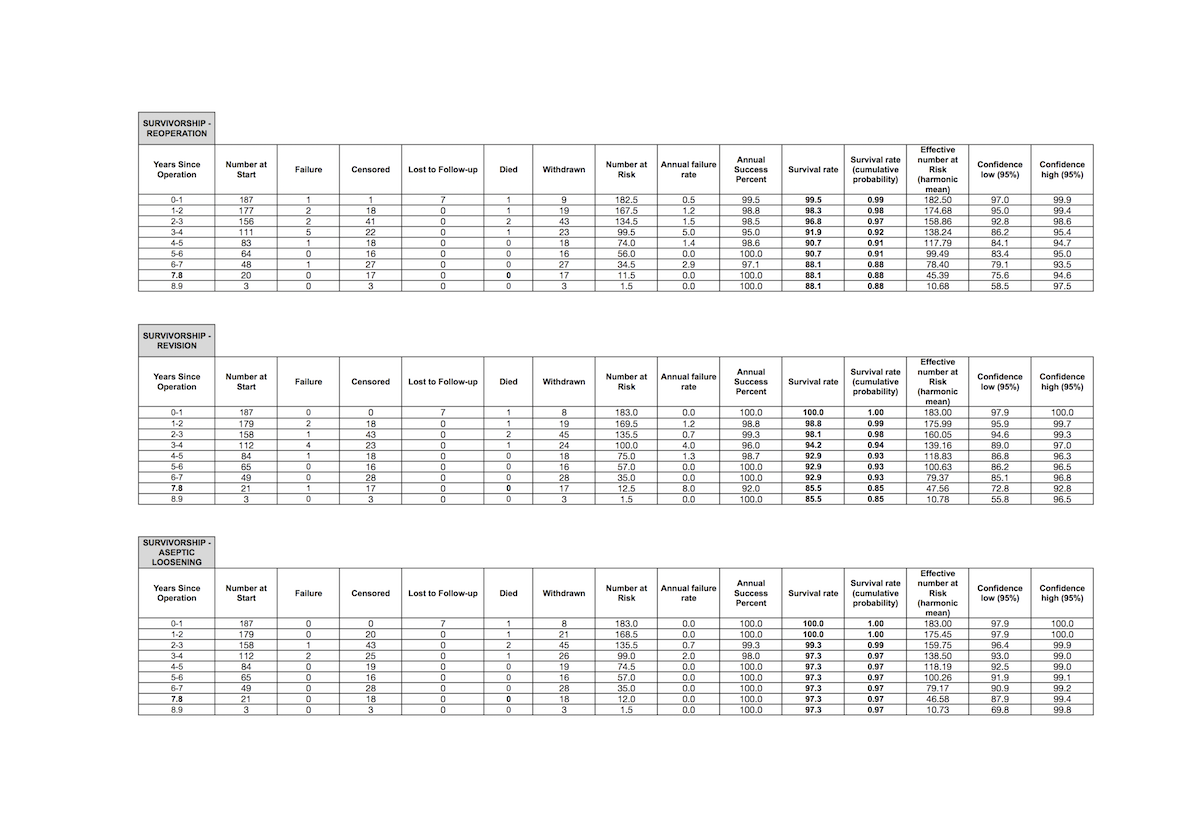

Supplement: Supplementary file 1 — Outcome life tables. (TIFF 3853 kb) [file 12891_2018_2099_MOESM1_ESM.tiff]
